# Prognostic ^18^F-flotufolastat PET parameters for outcome assessment of ^177^Lu-labeled PSMA-targeted radioligand therapy in metastatic castration-resistant prostate cancer

**DOI:** 10.1007/s00259-024-07003-2

**Published:** 2025-01-23

**Authors:** Amir Karimzadeh, Kimberley Hansen, Ergela Hasa, Bernhard Haller, Matthias M. Heck, Robert Tauber, Calogero D`Alessandria, Wolfgang A. Weber, Matthias Eiber, Isabel Rauscher

**Affiliations:** 1 Department of Nuclear Medicine, School of Medicine, Technical University of Munich, Munich, Germany; 2 Department of Diagnostic and Interventional Radiology and Nuclear Medicine, University Medical Center Hamburg-Eppendorf, Martinistr. 52, 20246 Hamburg, Germany; 3 Institute of AI and Informatics in Medicine, School of Medicine, Technical University of Munich, Munich, Germany; 4 Department of Urology, School of Medicine, Technical University of Munich, Munich, Germany; 5 Bavarian Cancer Research Center, Munich, Germany

**Keywords:** mCRPC, [^177^Lu]Lu-PSMA-I&T, ^18^F-flotufolastat, PSMA, Radioligand therapy

## Abstract

**Purpose:**

This retrospective analysis evaluates baseline ^18^F-flotufolastat positron emission tomography (PET) parameters as prognostic parameters for treatment response and outcome in patients with metastatic castration-resistant prostate cancer (mCRPC) undergoing treatment with [^177^Lu]Lu-PSMA-I&T.

**Methods:**

A total of 188 mCRPC patients with baseline ^18^F-flotufolastat PET scans were included. Tumor lesions were semiautomatically delineated, with imaging parameters including volume-based and standardized uptake value (SUV)-based metrics. Outcome measures included prostate-specific antigen (PSA) response, PSA-progression-free survival (PSA-PFS), and overall survival (OS). Univariate and multivariate regression analyses assessed the impact of baseline imaging and pretherapeutic clinical parameters on outcome. Event time distributions were estimated with the Kaplan-Meier method, and groups were compared with log-rank tests.

**Results:**

Significant prognostic parameters for PSA response and PSA-PFS included log-transformed whole-body SUVmax (odds ratio (OR), 3.26, 95% confidence interval (CI), 2.01–5.55 and hazard ratio (HR), 0.51, 95% CI, 0.4–0.66; both *p* < 0.001) and prior chemotherapy (OR 0.3, 95% CI, 0.12–0.72 and HR 1.64, 95% CI, 1.07–2.58; *p* = 0.008 and *p* = 0.028, respectively). For OS, significant prognosticators were the following log-transformed parameters: number of lesions (HR 1.38, 95% CI, 1.24–1.53; *p* < 0.001), TTV (HR 1.27, 95% CI, 1.18–1.37; *p* < 0.001), and ITLV (HR 1.24, 95% CI, 1.16–1.33; *p* < 0.001), with log-transformed TTV (HR 1.15, 95% CI, 1.04–1.27; *p* = 0.008) remaining significant in multivariate analysis.

**Conclusion:**

At baseline, SUV-based ^18^F-flotufolastat PET metrics (e.g., whole-body SUVmax) serve as significant positive prognosticators for short-term outcomes (PSA response and PSA-PFS). In contrast, volume-based metrics (e.g., TTV) are significant negative prognosticators for long-term outcome (OS), in mCRPC patients treated with [^177^Lu]Lu-PSMA-I&T.

**Supplementary Information:**

The online version contains supplementary material available at 10.1007/s00259-024-07003-2.

## Introduction

^177^Lu-labeled prostate-specific membrane antigen (PSMA)-targeted radioligand therapy (RLT) has emerged as an established treatment option for patients with metastatic castration-resistant prostate cancer (mCRPC). Initially supported by data from compassionate use programs, subsequent phase II and III trials have confirmed the low toxicity and therapeutic efficacy of ^177^Lu-labeled PSMA-RLT, with the first agent now approved by regulatory bodies worldwide [1–6]. Substudies from both the TheraP and the VISION trials demonstrated the prognostic value of baseline [^68^Ga]Ga-PSMA-11 positron emission tomography (PET), showing that a higher whole-body mean standardized uptake value (SUVmean) correlates with better treatment efficacy of [^177^Lu]Lu-PSMA-617 [7, 8]. These observations are supported by further analyses utilizing [^68^Ga]Ga-PSMA-11 PET, demonstrating that higher tumor volume and lower PSMA-ligand uptake intensity prognosticate poorer overall survival (OS) in patients treated with [^177^Lu]Lu-PSMA-617 [9–12]. Meanwhile, diagnostic imaging is increasingly shifting from ^68^Ga- to ^18^F-labeled PSMA-ligands due to several advantages of these compounds. These include a longer half-life and higher positron yield with lower energy, which contribute to higher diagnostic accuracy, as well as wider availability through facilitated delivery [13]. This shift has emphasized the prognostic impact of ^18^F-labeled PSMA-ligands (e.g., ^18^F-PSMA-1007, or ^18^F-flotufolastat (formerly ^18^F-rhPSMA-7.3) for mCRPC patients undergoing ^177^Lu-labeled PSMA-RLT. In response, a recent analysis reported that baseline tumor uptake of ^18^F-PSMA-1007 PET was significantly associated with outcome in mCRPC [14]. However, to date there are no data on the prognostic impact of ^18^F-flotufolastat, which was approved by the US Food and Drug Administration in May 2023 for diagnostic imaging of patients with suspected recurrent, or primary, prostate cancer [15]. Therefore, this retrospective analysis aimed to evaluate baseline ^18^F-flotufolastat PET parameters (e.g., total tumor volume (TTV), whole-body SUVmax) as potential prognostic parameters for prostate-specific antigen (PSA) response, PSA-progression-free survival (PSA-PFS) and OS in a large cohort of mCRPC patients receiving ^177^Lu-labeled PSMA-RLT.

## Materials and methods

### Patients and 177Lu-labeled PSMA-RLT

In this retrospective, single-center analysis data from patients with mCRPC undergoing [^177^Lu]Lu-PSMA-I&T therapy at our clinic between November 2017 and September 2021 were retrospectively reviewed. The patients received RLT with a standard activity of approximately 7.4 GBq [^177^Lu]Lu-PSMA-I&T at a median interval of 6 weeks. The treatment activity could be slightly adopted based on e.g. lab tests and tumor burden. Detailed patient characteristics are given in Table 1. Baseline ^18^F-flotufolastat PET scans were available for all patients. For treatment eligibility, PSMA-ligand uptake in tumor lesions had to be at least as high as the liver background. [^177^Lu]Lu-PSMA-I&T was prepared in accordance with good manufacturing practices and the German Medicinal Products Act (AMG § 13 2b). All patients gave written informed consent. Treatment was performed under the conditions outlined in article 37 of the Declaration of Helsinki concerning unproven interventions in clinical practice. This retrospective analysis was approved by the institutional ethics committee (reference number 115/18S).

**Table 1:** Patient characteristics

Characteristic		
**Age (yr)**	74 (68–79)	
**Time since initial diagnosis (yr)**	5.4 (3.4–9.5)	
**No. of [** ^**177**^ **Lu]Lu-PSMA-I&T cycles**	4 (2–6)	
**Pretherapeutic blood parameters**		
PSA, ng/mL (*n* = 184)	71.5 (23.0-219.4)	
LDH, U/L (*n* = 183)	251 (215-324.5)	
AP, U/L (*n* = 182)	117 (73.3-213.3)	
Hb, g/dL (*n* = 186)	11.9 (10.4–13.1)	
**Prior systemic therapies**		
Abiraterone	159 (84.6)	
Enzalutamide	115 (61.2)	
^223^Ra	15 (8.0)	
Docetaxel	131 (69.7)	
Cabazitaxel	25 (13.3)	
Previous chemotherapy	133 (70.7)	
**Site of metastasis**		
Lymph nodes	127 (67.6)	
Bone	174 (92.6)	
Visceral, overall	39 (20.7)	
Liver	13 (6.9)	
Lung/Pleura	20 (10.6)	
Adrenal	10 (5.3)	
Brain	2 (1.1)	
**Baseline** ^**18**^ **F-flotufolastat PET parameters**		
Number of lesions (n)	128 (41.0-239.0)	
TTV, mL	394.1 (122.2-1125.9)	
ITLV, mL	877.6 (255.7-2527.7)	
Highest SUV_max_	60.6 (36.9–98.0)	
Whole-body SUV_max_	13.9 (9.8–22.0)	
Whole-body SUV_mean_	5.6 (4.8–7.2)	
Whole-body SUV_peak_	9.0 (6.3–14.0)	

Data are reported as median (interquartile range) or n (%).^177^Lu = Lutiteum-177; PSMA = prostate-specific membrane antigen; PSA = prostate-specific antigen; LDH = lactate dehydrogenase; AP = alkaline phosphatase; Hb = hemoglobin; mCRPC = metastatic castration-resistant prostate cancer; ^223^Ra = Radium-223; ^18^F = Fluorine-18; PET = positron emission tomography; TTV = total tumor volume; ITLV = intensity-weighted lesion volume; SUV_max_ = maximum standardized uptake value; SUV_mean_ = mean standardized uptake value; SUV_peak_ = peak standardized uptake value

### PSMA-ligand PET imaging procedure

The radiolabeling of ^18^F-flotufolastat was carried out as previously described [16]. The preparation of ^18^F-flotufolastat adhered to the German Medicinal Products Act, AMG § 13 2b. All patients gave written informed consent. A mean activity of 309 ± 65 MBq of ^18^F-flotufolastat (range, 89–493 MBq) was administered via intravenous bolus, with scanning initiated at a median time of 70 min (interquartile range (IQR), 65–79 min) post-injection. Patients received a diluted oral contrast medium (300 mg of Telebrix; Guerbet) and 10 mg of furosemide. ^18^F-flotufolastat PET/computed tomography (CT) was performed on a Biograph mCT Flow scanner (Siemens Medical Solutions) or a Biograph Vision scanner (Siemens Medical Solutions). PET/CT scans were acquired in 3-dimensional mode with an acquisition time of 0.8 mm/s (mCT Flow scanner) and 1.1 mm/s (Vision scanner), respectively. PET images were reconstructed using ordered-subset expectation maximization (TrueX, 4 iterations, 8 subsets) followed by a postreconstruction smoothing Gaussian filter (3 mm in full width at half maximum). A diagnostic CT scan was initially performed in the portal venous phase 80 s after intravenous injection of an iodinated contrast agent (Imeron 300; Bracco Imaging) and was followed by the PET scan.

### Baseline 18F-flotufolastat parameters

The following imaging parameters were analyzed: number of lesions, TTV, intensity-weighted total lesion volume (ITLV = ∑ (lesion index × lesion uptake volume), highest SUVmax, whole-body SUVmax, SUVmean, and SUVpeak. These parameters were assessed through semiautomatic delineation using aPROMISE software [17–19]. Missed pathological foci were manually added when necessary, and PSMA-avid foci resulting from physiological tracer accumulation were removed. An experienced PSMA-ligand PET reader (IR) subsequently reviewed all lesions.

### Pretherapeutic clinical parameters, PSA response and PSA-progression-free survival

The following pretherapeutic clinical parameters were assessed: age, time since initial diagnosis, prior chemotherapy, the presence of liver metastases and visceral metastases (obtained from baseline ^18^F-flotufolastat) and PSA, alkaline phosphatase (AP), lactate dehydrogenase (LDH) and hemoglobin (Hb). PSA response was defined as PSA decline ≥ 50% from baseline according to Prostate Cancer Clinical Trials Working Group 3 [20]. PSA progression was either defined as PSA increase ≥ 25% and ≥ 2 ng/mL above the nadir after initial PSA decline or PSA increase ≥ 25% and ≥ 2 ng/mL from baseline in case with no PSA decline [20].

### Statistical analysis

Continuous covariates are reported as median values with IQR, while categorical covariates are described by their frequencies and proportions. Outcome measures are PSA response, PSA-PFS, and OS. The Kaplan-Meier method was used to estimate event time distributions, and log-rank tests were used to compare groups. Spearman’s rank correlation coefficient was used to quantify strength of association between quantitative variables. A logarithmic transformation (base 2) was applied to normalize data distribution and reduce skewness for the following parameters: number of lesions, TTV, ITLV, highest SUVmax, whole-body SUVmax, SUVmean and SUVpeak, time since initial diagnosis, PSA, AP, and LDH. Univariate and multivariate logistic and Cox regression analyses were performed to assess the impact of baseline imaging and pretherapeutic clinical parameters on PSA response, PSA-PFS, and OS. Highly correlated (*r* > 0.7; Supplementary Table 1) volume- and SUV-based metrics (highest SUVmax, whole-body SUVmax, and SUVmean as well as number of lesions and TTV) were included in multivariate logistic and Cox regression models and a backward variable selection procedure with a significance level of 0.05 was performed to determine the covariate(s) with the strongest association to the outcome variables. The remaining significant parameters were then included in a multivariate model together with other significant pretherapeutic clinical parameters from the univariate analyses. Whole-body SUVpeak and ITLV were excluded from this analysis due to their high correlation with whole-body SUVmax and TTV, respectively. Both indicating an almost perfect linear correlation and therefore providing no additional prognostic benefit (Supplementary Fig. 1A and B and Supplementary Table 1). Odds ratios (OR), hazard ratios (HR) and corresponding 95% confidence intervals (CI) are presented, with a *p*-value of < 0.05 considered statistically significant. Statistical analyses were performed using GraphPad Prism version 10.2.2 (341) for Mac.

## Results

Median follow-up time was 13.3 months (IQR, 6.7–21.6 months). PSA response was achieved in 36.1% (65/180) patients. Median OS was 14.4 months (95% CI, 12.9–15.9 months) and median PSA-PFS was 4.1 months (95% CI, 3.2–5.0 months). At the time of analysis, 74.5% (140/188) patients had shown PSA progression and 86.2% (162/188) patients had deceased.

### PSA response and PSA-progression-free survival

Detailed results of univariate and multivariate logistic and Cox regression analyses for PSA response and PSA-PFS are presented in Tables 2 and 3, respectively. Baseline imaging parameters that were significant prognosticators of PSA response and PSA-PFS in the univariate analyses include the following log-transformed SUV-based metrics: highest SUVmax (OR 1.85, 95% CI, 1.33–2.62 and HR 0.68, 95% CI, 0.57–0.81; both *p* < 0.001), whole-body SUVmax (OR 2.80, 95% CI, 1.83–4.48 and HR, 0.59, 95% CI, 0.47–0.74; both *p* < 0.001), whole-body SUVmean (OR, 3.96, 95% CI, 2.0-8.33 and HR. 0.60, 95% CI, 0.42–0.86; *p* < 0.001 and *p* = 0.006, respectively), and whole-body SUVpeak (OR, 2.65, 95% CI, 1.71–4.25 and HR, 0.6, 95% CI, 0.47–0.76; both *p* < 0.001). A stepwise backward multivariate logistic and Cox regression analysis was performed, identifying whole-body SUVmax as the strongest prognosticator for both PSA response and PSA-PFS. Whole-body SUVmax was then tested against all other significant pretherapeutic clinical parameters from the univariate analysis, namely age, log-transformed time since initial diagnosis, prior chemotherapy, log-transformed AP (only for PSA-PFS) and log-transformed LDH. In this multivariate analysis, whole-body SUVmax remained a significant prognosticator for PSA response (OR, 3.26, 95% CI, 2.01–5.55, *p* < 0.001) and PSA-PFS (HR, 0.51, 95% CI, 0.4–0.66, *p* < 0.001). Prior chemotherapy also showed significance as a prognosticator for both PSA response (OR, 0.3, 95% CI, 0.12–0.72, *p* = 0.008) and PSA-PFS (HR, 1.64, 95% CI, 1.07–2.58, *p* = 0.028).

**Table 2:** Logistic regression analysis of baseline parameters and PSA Response

Parameter	Univariate logistic regression, OR (95%CI)		*p*-value	Multiple logistic regression, OR (95%CI)	*p*-value					
**PSMA-ligand PET parameters**										
Number of lesions		0.95 [0.8–1.15]	0.611							
TTV		1.02 [0.89–1.16]	0.802							
ITLV		1.05 [0.92–1.19]	0.488							
Highest SUV_max_		1.85 [1.33–2.62]	**< 0.001**							
Whole-body SUV_max_		2.80 [1.83–4.48]	**< 0.001**	3.26 [2.01–5.55]	**< 0.001**					
Whole-body SUV_mean_		3.96 [2.0-8.33]	**< 0.001**							
Whole-body SUV_peak_		2.65 [1.71–4.25]	**< 0.001**							
**Pretherapeutic clinical parameters**										
Age		1.04 [1.0-1.08]	**0.035**	1.0 [0.95–1.06]	0.987					
Time since initial diagnosis		1.38 [1.03–1.87]	**0.034**	1.31 [0.9–1.94]	0.166					
Prior chemotherapy		0.33 [0.17–0.64]	**0.001**	0.3 [0.12–0.72]	**0.008**					
Liver metastases		1.12 [0.32–3.49]	0.855							
Visceral metastases		0.95 [0.44-2.0]	0.890							
PSA		1.02 [0.90–1.15]	0.810							
AP		0.78 [0.58–1.03]	0.091							
LDH		0.57 [0.31–0.93]	**0.039**	0.79 [0.42–1.37]	0.435					
Hb		1.15 [0.98–1.37]	0.094							

All continuous parameters except age and Hb were log (base2) transformed. Significant *p*-values are given in bold. PSA = prostate-specific antigen; OR = odds ratio; CI = confidence interval; PSMA = prostatespecific membrane antigen; PET = positron emission tomography; TTV = total tumor volume; ITLV = intensity-weighted lesion volume; SUV_max_ = maximum standardized uptake value; SUV_mean_ = mean standardized uptake value; SUV_peak_ = peak standardized uptake value; AP = alkaline phosphatase; LDH = lactate dehydrogenase; Hb = hemoglobin

**Table 3:** Cox Regression Analysis of Baseline Parameters and PSA-PFS

Parameter	Univariate Cox regression, HR (95%CI)	*p*-value	Multivariate Cox regression, HR (95%CI)	*p*-value
**PSMA-ligand PET parameters**				
Number of lesions	1.04 [0.93–1.15]	0.529		
TTV	0.99 [0.92–1.06]	0.722		
ITLV	0.97 [0.91–1.04]	0.436		
Highest SUV_max_	0.68 [0.57–0.81]	**< 0.001**		
Whole-body SUV_max_	0.59 [0.47–0.74]	**< 0.001**	0.51 [0.4–0.66]	**< 0.001**
Whole-body SUV_mean_	0.60 [0.42–0.86]	**0.006**		
Whole-body SUV_peak_	0.6 [0.47–0.76]	**< 0.001**		
**Pretherapeutic clinical parameters**				
Age	0.96 [0.94–0.98]	**< 0.001**	0.98 [0.96–1.01]	0.226
Time since initial diagnosis	0.8 [0.68–0.93]	**0.005**	0.9 [0.74–1.09]	0.266
Prior chemotherapy	1.68 [1.15–2.49]	**0.008**	1.64 [1.07–2.58]	**0.028**
Liver metastases	1.0 [0.52–1.74]	0.986		
Visceral metastases	0.80 [0.52–1.19]	0.292		
PSA	1.02 [0.96–1.09]	0.543		
AP	1.27 [1.09–1.47]	**0.002**	1.09 [0.89–1.33]	0.387
LDH	1.46 [1.18–1.76]	**< 0.001**	1.30 [0.98–1.69]	0.056
Hb	0.50 [0.23–1.11]	0.084		

All continuous parameters except age and Hb were log (base2) transformed. Significant p*-*values are given in bold. PSA = prostate-specific antigen; PFS = progression-free survival; HR = hazard ratio; CI = confidence interval; PSMA = prostate-specific membrane antigen; PET = positron emission tomography; TTV = total tumor volume ITLV = intensity-weighted lesion volume; SUV_max_ = maximum standardized uptake value; SUV_mean_ = mean standardized uptake value; SUV_peak_ = peak standardized uptake value; AP = alkaline phosphatase; LDH = lactate dehydrogenase; Hb = hemoglobin

### Overall survival

**Fig. 1:**
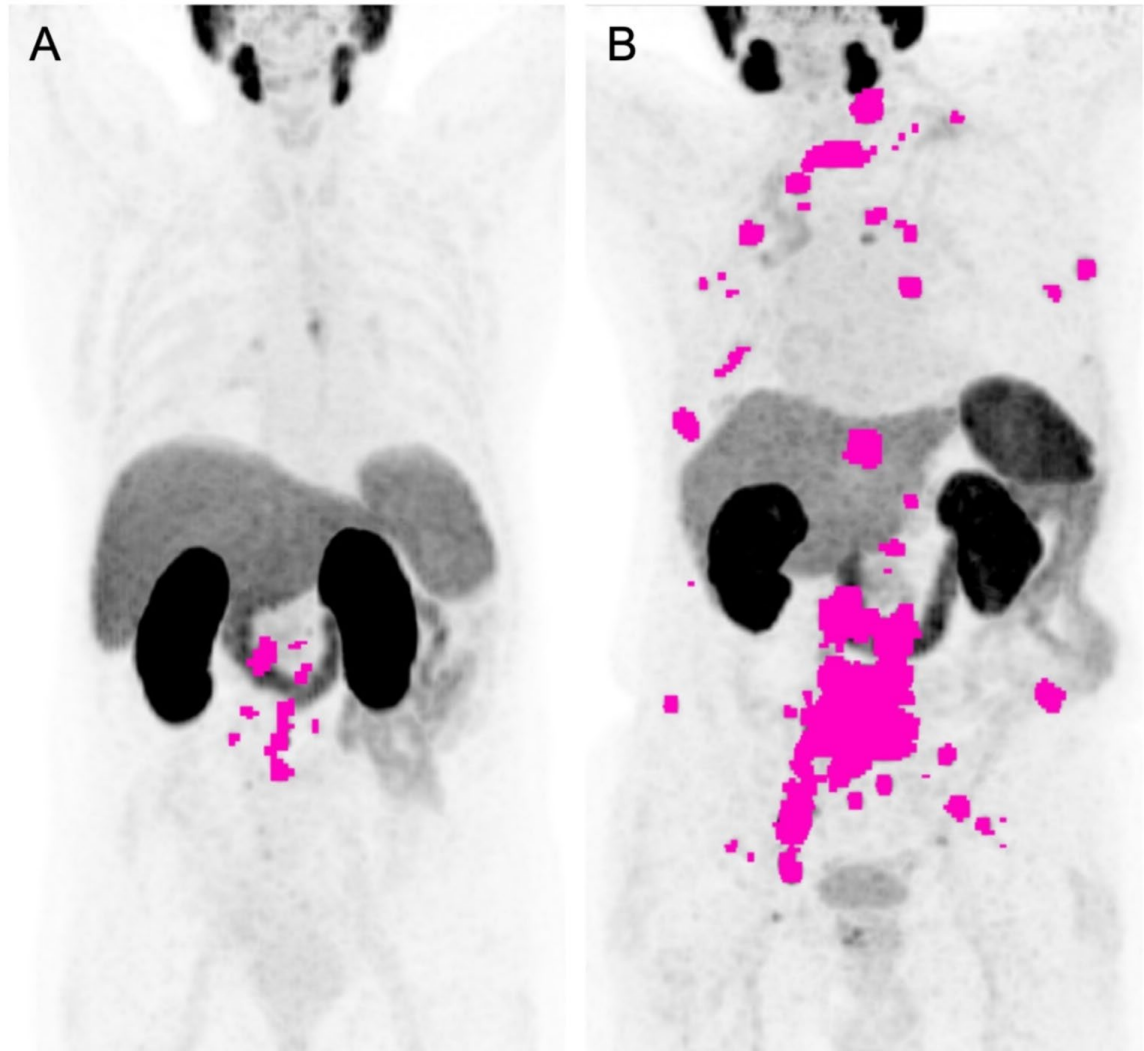
(**A**) A 70-year-old patient with lymph node metastases, showing 17 tumor lesions, a total tumor volume (TTV) of 31 ml, and an intensity-weighted total lesion volume (ITLV) of 55 ml. (**B**) A 77-year-old patient with bone metastases, showing 139 tumor lesions, a TTV of 322 ml, and an ITLV of 626 ml. PSA-PFS and OS were 5.4 months and 54.9 months for patient A, compared to 1.8 months and 12.0 months for patient B. The number of lesions, TTV, and ITLV were semiautomatically assessed, with tumor lesions shown in pink

Detailed results for univariate and multivariate Cox regression analyses for OS are presented in Table 4. Baseline imaging parameters significantly prognostic for OS in univariate Cox regression analysis included the following log-transformed volume-based metrics: number of lesions (HR, 1.376, 95% CI, 1.24–1.533; *p* < 0.001), TTV (HR, 1.271, 95% CI, 1.182–1.368; *p* < 0.001), and ITLV (HR, 1.24, 95% CI, 1.158–1.329; *p* < 0.001). Figure 1 presents examples of two patients with varying levels of these parameters and different outcomes. A stepwise backward Cox regression analysis was performed, including the number of lesions and TTV as candidates, which identified TTV as the stronger prognosticator for OS. When testing the prognostic value of TTV adjusting for significant pretherapeutic clinical parameters from the univariate analysis, namely log-transformed time since initial diagnosis, prior chemotherapy, the presence of liver metastases and visceral metastases, log-transformed PSA, log-transformed AP, log-transformed LDH and Hb, the following remained significant prognosticators of OS: log-transformed TTV (HR, 1.147, 95% CI, 1.038–1.271; *p* = 0.008), presence of visceral metastases (HR, 1.764, 95% CI; 1.038–2.874; *p* = 0.028), log-transformed LDH (HR, 1.421, 95% CI, 1.032–1.921; *p* = 0.026), and Hb (HR, 0.86, 95% CI, 0.761–0.97; *p* = 0.015).

**Table 4:** Cox Regression Analysis of Baseline Parameters and OS

Parameter	Univariate Cox regression, HR (95%CI)	*p*-value	Multivariate Cox regression, HR (95%CI)	*p*-value
**PSMA-ligand PET parameters**				
Number of lesions	1.38 [1.24–1.53]	**< 0.001**		
TTV	1.27 [1.18–1.37]	**< 0.001**	1.15 [1.04–1.27]	**0.008**
ITLV	1.24 [1.16–1.33]	**< 0.001**		
Highest SUV_max_	1.12 [0.97–1.29]	0.139		
Whole-body SUV_max_	0.96 [0.79–1.17]	0.711		
Whole-body SUV_mean_	0.78 [0.55–1.09]	0.148		
Whole-body SUV_peak_	0.98 [0.81–1.19]	0.849		
**Pretherapeutic clinical parameters**				
Age	1.0 [0.98–1.02]	0.988		
Time since initial diagnosis	0.75 [0.65–0.88]	**< 0.001**	0.85 [0.71–1.02]	0.089
Prior chemotherapy	1.56 [1.11–2.23]	**0.012**	1.10 [0.75–1.65]	0.63
Liver metastases	2.07 [1.05–3.68]	**0.021**	0.89 [0.41–1.83]	0.747
Visceral metastases	1.77 [1.18–2.58]	**0.004**	1.76 [1.04–2.87]	**0.028**
PSA	1.17 (1.091–1.250)	**< 0.001**	0.98 [0.9–1.07]	0.684
AP	1.56 (1.365–1.779)	**< 0.001**	1.14 (0.941–1.367)	0.173
LDH	2.21 (1.801–2.651)	**< 0.001**	1.42 (1.032–1.921)	**0.026**
Hb	0.7 (0.631–0.774)	**< 0.001**	0.86 (0.761–0.907)	**0.015**

All continuous parameters except age and Hb were log (base2) transformed. Significant *P-*values are given in bold. OS = overall survival; HR = hazard ratio; CI = confidence interval; PSMA = prostatespecific membrane antigen; PET = positron emission tomography; TTV = total tumor volume; ITLV = intensity-weighted lesion volume; SUV_max_ = maximum standardized uptake value; SUV_mean_ = mean standardized uptake value; SUV_peak_ = peak standardized uptake value, PSA = prostate-specific antigen; AP = alkaline phosphatase; LDH = lactate dehydrogenase; Hb = hemoglobin

### Risk stratification model

**Fig. 2:**
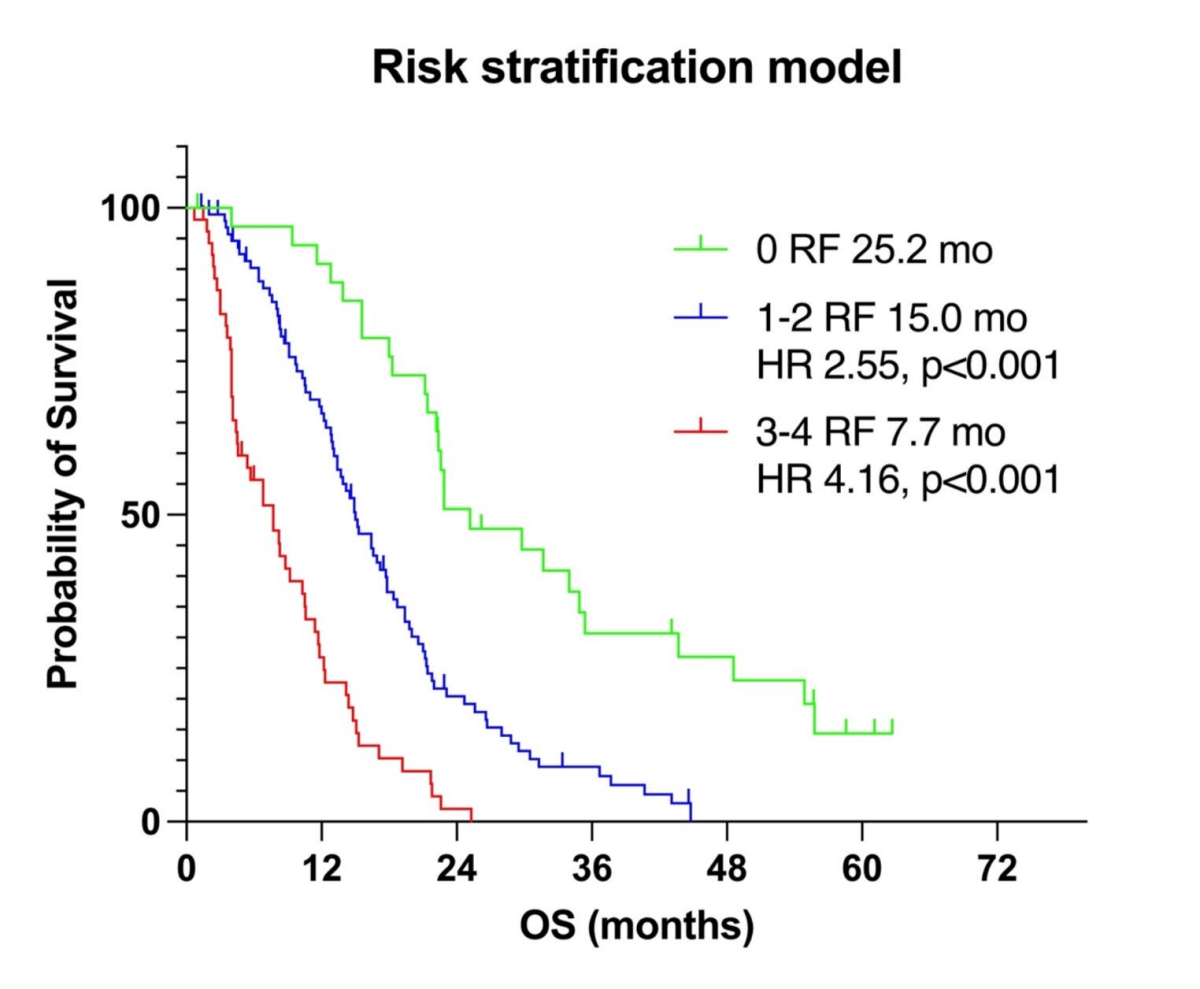
Kaplan-Meier survival curves for the risk stratification model, which includes baseline imaging and pretherapeutic clinical parameters that reached significance in the multivariate Cox regression analysis for OS. The model consists of the upper median values for total tumor volume (TTV) and lactate dehydrogenase (LDH) levels, the lower median value for hemoglobin (Hb), and the presence of visceral metastases. Each parameter represents one risk factor (RF). Patients were stratified based on the number of RFs: 0 RF (green line), 1–2 RFs (blue line), and 3–4 RFs (red line)

Following the approach of Hartrampf et al. [14], we propose a risk factor (RF) stratification model including all parameters that reached significance in multivariate Cox regression analysis for OS (Fig. 2). The model included high TTV (> 394.1 mL) and LDH levels (> 251 U/L) defined as values above the median, low Hb levels (< 11.9 g/dL) defined as values below the median, and the presence of visceral metastases, with each parameter representing one RF. Patients with no RFs had a median OS of 25.2 months. Patients with 1–2 RFs had a median OS of 15.0 months with a HR of 2.55 (*p* < 0.001) compared to those with 0 RFs. Patients with 3–4 RFs had a median OS of 7.7 months with an HR of 4.16 (*p* < 0.001) compared to those with 0 RFs.

## Discussion

SUV-based metrics in baseline ^18^F-flotufolastat PET prior to [^177^Lu] Lu-PSMA-I&T, including highest SUVmax, whole-body SUVmax, whole-body SUVmean, and whole-body SUVpeak, were significantly associated with PSA response and PSA-PFS in univariate analysis. Among these, whole-body SUVmax remained a significant prognosticator for both PSA response and PSA-PFS in multivariate analysis. However, these imaging parameters were not significant prognosticators of OS. In contrast, for OS, significant prognosticators included volume-based metrics such as the number of lesions, TTV, and ITLV with TTV remaining significant in multivariate analysis. The combination of imaging and pretherapeutic clinical parameters, namely TTV, the presence of visceral metastases, LDH, and Hb levels, that remained significant prognosticators of OS in multivariate analysis enabled effective stratification of patients regarding survival.

Despite the scarcity of comparative studies, our findings showed consistency with existing data on ^18^F-labeled compounds in patients with mCRPC receiving ^177^Lu-labeled PSMA-RLT. Specifically, our results confirm aspects of a recent analysis by Hartrampf et al., which explored the prognostic value of baseline ^18^F-PSMA-1007 PET-derived parameters in mCRPC patients undergoing [^177^Lu] Lu-PSMA-I&T therapy [14]. Consistent with the prior analysis we found that volume-based metrics, such as PSMA-positive tumor volume, showed no significant impact on prognosticating PSA response [14]. We also confirm that whole-body SUVmean has a significant prognostic impact on PSA response, with a OR of 3.96 (*p* < 0.001) in our analysis compared to 1.18 (*p* = 0.004) in Hartrampf et al. [14]. However, due to a logarithmic transformation of SUV-based metrics in our analysis, direct comparison between the OR is not possible. Interestingly, in our analysis, log-transformed whole-body SUVmax was an even better prognosticator of PSA response (OR 2.80, *p* < 0.001 vs. OR 1.00, *p* = 0.27 in [14]), and it also significantly prognosticated PSA-PFS (HR 0.59, *p* < 0.001). Our findings on the impact of SUV-based metrics on PSA response and PSA-PFS are also consistent with recent analyses using [^68^Ga] Ga-PSMA-11 PET as baseline imaging before ^177^Lu-labeled PSMA-RLT [7, 9, 12], further underlying their potential role in prognosticating short-term outcomes. Given that the calculation of whole-body SUVmean is more complex and depends heavily on the segmentation method, SUVmax measurements are easier to implement in clinical routine. However, SUVmax, representing the highest voxel value within the region of interest, is susceptible to image noise, which can lead to variability in measurements, particularly in small lesions, where noise may significantly affect the maximum voxel value. Additionally, if a lesion is not precisely centered within the voxel, SUVmax may underestimate true activity, misrepresenting the lesions intensity [21]. Despite these potential drawbacks, we believe that measuring the mean SUVmax across the entire tumor burden, as represented by whole-body SUVmax, may help reduce these weaknesses and improve the reliability of SUVmax as a prognostic parameter. In contrast, SUVpeak includes a local average of SUV values from surrounding voxels, which reduces noise impact and provides a more stable, statistically reliable measurement. However, this averaging process can make SUVpeak less representative of the true activity, particularly in small lesions, compared to SUVmax. Additionally, the lack of standardization of SUVpeak across imaging protocols can result in inconsistencies in its application and interpretation [21].

While Hartrampf et al. [14] demonstrated a significant impact of whole-body SUVmean on OS (HR, 0.91; *p* = 0.03), our data showed no significant association between log-transformed SUV-based metrics in general, and particularly whole-body SUVmean with OS (HR, 0.78; *p* = 0.148). The positive association between whole-body SUVmean and OS has also been thoroughly reported in the context of [^68^Ga] Ga-PSMA-11 PET [7–10, 12]. For instance, a VISION trial substudy found that higher baseline SUVmean was associated with better OS (HR, 0.88), with the highest quartile having an OS of 21.4 months vs. 12.6–14.6 months in the lower quartiles [8]. The median whole-body SUVmean in our cohort was 5.6 with an IQR of 4.8–7.2, indicating more homogeneous tumor uptake, which may explain the lack of correlation with OS. This reduced variability and effect size potentially reflect a narrower range of tumor differentiation, which could reduce the prognostic impact of whole-body SUVmean. However, further analyses are necessary to confirm this hypothesis and to assess the usability of SUV-based metrics in mCRPC with ^18^F-flotufolastat PET prior to ^177^Lu-labeled PSMA-RLT, especially considering the ongoing shift from ^68^Ga- to ^18^F-labeled PSMA ligands.

Our analysis shows a significant association between OS and all volume-based metrics, with TTV demonstrating significant prognostic value in multivariate analysis. These findings are in line with other retrospective analyses using [^68^Ga] Ga-PSMA-11 PET prior to ^177^Lu-labeled PSMA-RLT [10, 11] and comparable to the results from Seifert et al. [10], who reported a significant impact of log-transformed number of lesions (HR, 1.255; *p* = 0.009 vs. HR, 1.38; *p* < 0.001 in our analysis) and log-transformed PSMA-TV (HR, 1.299; *p* = 0.005 vs. HR, 1.27; *p* < 0.001 in our analysis). Our results underline the potential of volume-based metrics as a prognostic marker for long-term outcome.

In addition to baseline ^18^F-flotufolastat PET parameters, pretherapeutic clinical parameters significantly impacted prognosticating outcome. Prior chemotherapy was significantly associated with worse short-term outcomes like PSA response and PSA-PFS in multivariate analyses (OR, 0.3 and HR, 1.64; *p* = 0.008 and *p* = 0.028, respectively). This association, previously reported with ^177^Lu-labeled PSMA-RLT and radiographic PFS, likely reflects the more advanced disease stage of heavily pretreated patients [12,22,23]. The same applies to the role of visceral metastases, where their presence at the start of ^177^Lu-labeled PSMA-RLT indicates more aggressive disease and poorer outcomes. Numerous retrospective analyses have shown their negative prognostic impact on OS [2, 6, 12]. Our findings also demonstrate that visceral metastases are significantly associated with poorer OS (HR, 1.76, *p* = 0.028). Furthermore, our results demonstrate that higher levels of log-transformed LDH and lower levels of Hb are independently associated with poorer OS (HR, 1.42; *p* = 0.026; and HR, 0.86; *p* = 0.015, respectively). These associations were also reported by previous retrospective analyses [2, 6, 12], further underlying the prognostic significance of these parameters.

The combination of all significant prognostic parameters of OS from the multivariate analysis into a risk stratification model—including high tumor volume (TTV > 394.1 mL), elevated lactate dehydrogenase (LDH > 251 U/L), low hemoglobin (Hb < 11.9 g/dL), and the presence of visceral metastases—allowed for effective stratification of outcomes. Patients with 1–2 RFs had an approximately two times higher risk of death (HR 2.546; *p* < 0.001), and those with 3–4 RFs had an approximately four times higher risk of death (HR 4.161; *p* < 0.001) compared to those with 0 RFs. This risk stratification model demonstrates that combining risk factors identified in our multivariate analysis provides a potential tool for prognosticating outcomes.

In addition to the retrospective nature of the present analysis, this analysis has several limitations. Volume-based metrics were assessed without distinguishing the origin of lesions, which could affect outcomes. Additionally, as mentioned above, the SUVmean calculation depends on the segmentation method, unlike parameters such as SUVmax, which should be considered when comparing with other studies using different approaches (e.g., relative vs. fixed thresholding). A further limitation of this analysis is the slightly wider imaging window with a median time of 70 min post-injection compared to the 60-minute timeframe recommended by EANM guidelines [24] and the 50–70-minute window used in the Phase III LIGHTHOUSE and SPOTLIGHT studies [25,26], which could introduce minor variability in tracer uptake quantification.

## Conclusion

In conclusion, baseline ^18^F-flotufolastat PET prior to ^177^Lu-labeled PSMA-RLT demonstrated significant prognostic value for outcome. SUV-based metrics, such as whole-body SUVmax, were useful for prognosticating short-term outcome (PSA response and PSA-PFS), while volume-based metrics (e.g., TTV) showed utility for prognosticating long-term outcomes (OS).

## Electronic supplementary material

Below is the link to the electronic supplementary material.

Supplementary Material 1

## Data Availability

The datasets supporting the conclusions of this study can be made available on reasonable request.

## Abbreviations

AP: Alkaline phosphatase
CI: Confidence interval
CT: Computed tomography
Hb: Hemoglobin
HR: Hazard ratio
IQR: Interquartile range
ITLV: Intensity-weighted total lesion volume
LDH: Lactate dehydrogenase
mCRPC: Metastatic castration-resistant prostate cancer
OR: Odds ratio
OS: Overall survival
PET: Positron emission tomography
PFS: Progression-free survival
PSA: Prostate-specific antigen
PSMA: Prostate-specific membrane antigen
RF: Risk factor
RLT: Radioligand therapy
SUV: Standardized uptake value
TTV: Total tumor volume
