# Supplementary material for: Prognostic 18F-flotufolastat PET parameters for outcome assessment of 177Lu-labeled PSMA-targeted radioligand therapy in metastatic castration-resistant prostate cancer
Source: Eur J Nucl Med Mol Imaging. 2025 Jan 23;52(6):2041–50. doi: 10.1007/s00259-024-07003-2 (PMC12014739; doi:10.1007/s00259-024-07003-2)
Supplement: Supplementary file 1 — Supplementary Material 1 [file 259_2024_7003_MOESM1_ESM.docx]

**SUPPLEMENTARY TABLE 1.** Correlation of baseline ^18^F-Flotufolastat PET parameters

| **Parameter 1** | **Parameter 2** | **r** | **P value** |  |
| --- | --- | --- | --- | --- |
| Number of Lesions | TTV | 0.79 | <0.001 | |
| Number of Lesions | ITLV | 0.76 | <0.001 | |
| TTV | ITLV | 0.99 | <0.001 | |
| Highest SUVmax | Whole-body SUVmax | 0.76 | <0.001 | |
| Highest SUVmax | Whole-body SUVmean | 0.55 | <0.001 | |
| Highest SUVmax | Whole-body SUVpeak | 0.72 | <0.001 | |
| Whole-body SUVmax | Whole-body SUVmean | 0.81 | <0.001 | |
| Whole-body SUVmax | Whole-body SUVpeak | 0.97 | <0.001 | |
| Whole-body SUVmean | Whole-body SUVpeak | 0.8 | <0.001 | |
| Spearman’s rank correlations are shown. TTV = total tumor volume; ITLV = intensity-weighted lesion volume; SUV_max_ = maximum standardized uptake value; SUV_mean_ = mean standardized uptake value; SUV_peak_ = peak standardized uptake value. | | | | |


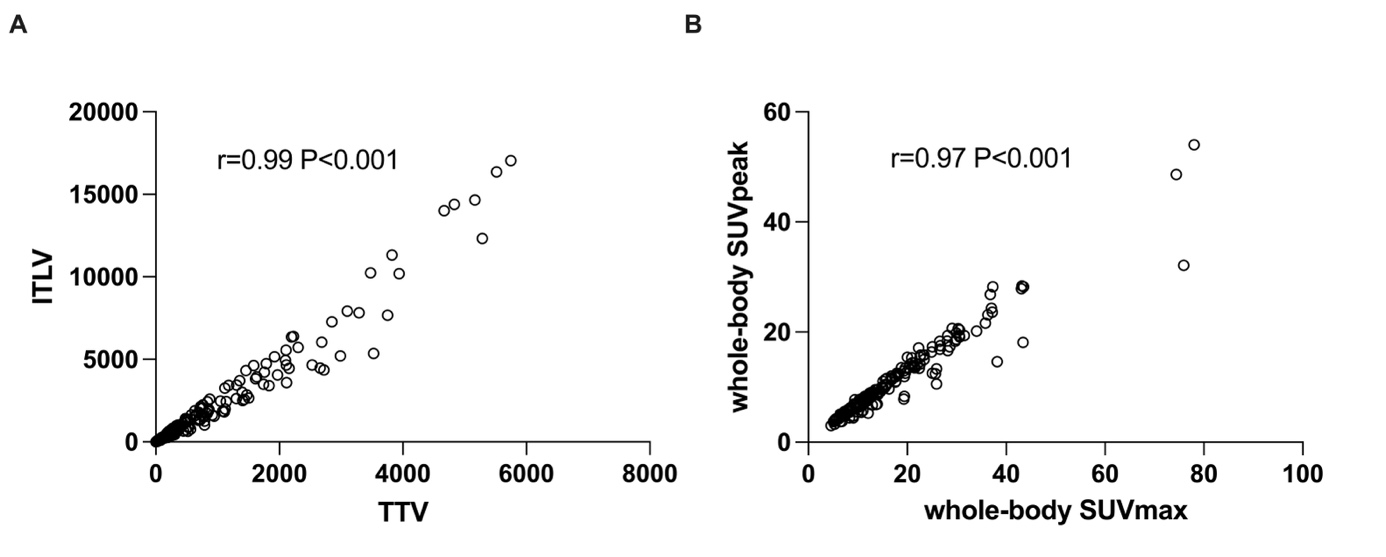


Supplementary Figure 1. Results of Spearman’s rank correlations for (A) TTV and ITLV, and (B) whole-body SUVmax and whole-body SUVpeak.
